# Evolving patterns in systemic treatment utilization and survival among older patients with advanced cutaneous melanoma

**DOI:** 10.1002/cam4.70131

**Published:** 2024-08-28

**Authors:** Yoon Duk Hong, Lindsey Enewold, Elad Sharon, Jeremy L. Warner, Amy J. Davidoff, Chris Zeruto, Angela B. Mariotto

**Affiliations:** ^1^ Division of Cancer Control and Population Sciences National Cancer Institute Bethesda Maryland USA; ^2^ Kelly Services, Inc. Rockville Maryland USA; ^3^ Division of Cancer Treatment & Diagnosis National Cancer Institute Bethesda Maryland USA; ^4^ Lifespan Cancer Institute, Rhode Island Hospital Providence Rhode Island USA; ^5^ Center for Clinical Cancer Informatics and Data Science, Legorreta Cancer Center Brown University Providence Rhode Island USA; ^6^ Information Management Services, Inc. Calverton Maryland USA

## Abstract

**Introduction:**

In the last decade, melanoma treatment has improved significantly. However, data on population‐level treatment utilization and survival trends among older patients is limited. This study aimed to analyze trends in systemic anticancer therapy (Rx), including the uptake of immune checkpoint inhibitors (ICIs), in conjunction with trends in cause‐specific survival among older patients (66+) diagnosed with advanced melanoma (2008–2019).

**Methods:**

We used the Surveillance, Epidemiology, and End Results (SEER)‐Medicare Condensed Resource to assess any Rx utilization among patients first diagnosed with advanced melanoma in 2008–2010, 2011–2014, and 2015–2019, stratified by stage, and type of first‐line Rx among patients receiving Rx. The SEER dataset was used to evaluate trends in cause‐specific survival by year of diagnosis.

**Results:**

Rx utilization (any type) almost doubled, from 28.6% (2008–2010) to 55.4% (2015–2019) for stage 3 melanoma, and from 35.5% to 68.0% for stage 4 melanoma. In 2008–2010, the standard first‐line treatment was cytokines/cytotoxic chemotherapy/other. By 2015–2019, only 5.1% (stage 3) and <3.6% (stage 4) of patients receiving Rx received these agents, as ICIs emerged as the dominant treatment. Both 1‐year and 5‐year cause‐specific survival significantly improved since 2010 for stage 4 and since 2013 for stage 3.

**Conclusions:**

This study shows a significant rise in Rx utilization and a rapid transition from cytokines/cytotoxic chemotherapy to ICIs, reflecting a rapid uptake of highly effective treatment in a previously challenging disease with limited options before 2011. The documented survival improvement aligns with the adoption of these novel treatments, underscoring their significant impact on real‐world patient outcomes.

## 1 INTRODUCTION

**FIGURE 1:**
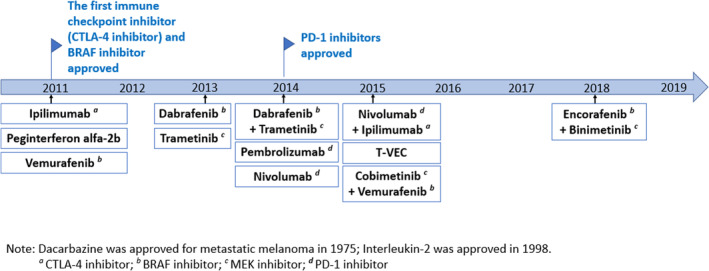
FDA approval of melanoma treatments (2011–2019).

For decades, there were limited systemic treatment options for advanced melanoma, with the mainstay of therapy being cytotoxic chemotherapy agents, such as dacarbazine, and cytokine‐based immunotherapy, such as interleukin‐2 (IL‐2). These treatments provided very limited survival benefits and were often accompanied by high toxicity.^1^, ^2^ Historically, older patients with melanoma experienced suboptimal clinical management and treatment, exhibiting lower rates of surgical and systemic treatment compared to their younger counterparts.^3^, ^4^, ^5^, ^6^ Since 2011, the treatment landscape for advanced melanoma has evolved rapidly, with the introduction of the first immune checkpoint inhibitor (ICI) and v‐raf murine sarcoma viral oncogene homolog B1 (BRAF) inhibitor in 2011, and mitogen‐activated protein kinase kinase (MEK) inhibitor in 2013. Programmed death‐1 (PD‐1) inhibitors were first approved in 2014. (Figure 1).

Given the increased availability of effective treatment options in this new era of melanoma treatment, it is important to understand how patterns of systemic treatment among older patients with advanced melanoma may have changed. It is also imperative to understand whether outcomes, such as survival, have changed over time for older patients with melanoma, alongside recent advances in treatment.

Previous studies have examined treatment patterns^7^ and differences in treatment receipt, specifically, immunotherapy receipt, across subgroups of patients diagnosed with melanoma.^8^, ^9^, ^10^, ^11^ However, to our knowledge, no studies have analyzed trends in survival in conjunction with the availability of new treatments. Hence, in this study, we aimed to assess (1) trends in any melanoma systemic treatment receipt, (2) factors associated with any melanoma systemic treatment receipt, (3) changes in the selection of first‐line systemic treatment over time among older patients with advanced melanoma, and (4) trends in cause‐specific survival, examining periods that cover both the pre‐ and post‐novel therapy eras.

## 2 METHODS

### 2.1 Treatment utilization analysis

#### 2.1.1 Data source

The Surveillance, Epidemiology and End Results (SEER)‐Medicare Condensed Resource (CoRe) (https://healthcaredelivery.cancer.gov/seermedicare/medicare/summarized‐data.html) was used for this cohort study to examine treatment utilization. The CoRe data files are a condensed version of the original SEER‐Medicare files^12^ which have been simplified to facilitate use of SEER‐Medicare data.^13^ Files used for the study included the SEER‐Medicare Cancer File, which has information related to the cancer diagnosis and demographics, the CoRe Enrollment File, which includes information on monthly enrollment in Medicare Parts A, B, C (Medicare Advantage) and/or D, the CoRe Prediagnosis Comorbidity File, which has flags for prediagnosis comorbid conditions and the Charlson and NCI Comorbidity Indices, and the CoRe Systemic Treatment AB and D Files, which summarize cancer systemic therapy provided under Medicare Parts A and B, and Part D, respectively.

#### 2.1.2 Study population

The SEER‐Medicare CoRe files are limited to patients who have a cancer diagnosis included in the SEER‐Medicare Cancer File and are continuously enrolled in Medicare Parts A and B fee‐for‐service (FFS) plans from 12 months prior to cancer diagnosis through at least 1 month after diagnosis (for inclusion in the Part ABFFS cohort) and/or Part D from 4 months prior to cancer diagnosis through at least 1 month after diagnosis (for inclusion in the Part D cohort). Patients remain in the cohort(s) until: (a) subsequent cancer diagnosis, (b) end of ABFFS/D coverage, (c) death, or (d) end of claims (for this analysis December 31, 2020), whichever occurs first. Patients are excluded from the SEER‐Medicare CoRe ABFFS cohort if diagnosed prior to 2000 and from the Part D cohort if diagnosed prior to 2008. As age‐related eligibility for Medicare begins at the age of 65 years and 12‐month prediagnosis data are used for baseline information, patients are excluded from both cohorts if age at diagnosis is <66 years or unknown. Patients are also excluded if the tumor is nonmalignant/malignancy unknown, the month of diagnosis is unknown, or if the cancer is diagnosed at autopsy or through a death certificate only.^13^

The analytic cohort for this study was further restricted to patients who were included in both ABFFS and D cohorts, i.e., enrolled in Medicare Parts A, B and D, and who had a diagnosis of stage 3 or 4 cutaneous melanoma (ICD‐O‐3 site: C440‐C449, ICD‐O‐3 histology: 8720–8790; first or only primary) between January 1, 2008 and December 31, 2019. Continuous inclusion in the ABFFS and D cohorts was required for at least 12 months following diagnosis (including the month of diagnosis) or until death, whichever occurred first, to ensure that patients' claim records were complete for treatment assessment. We also excluded patients with unknown or missing baseline patient variables.

#### 2.1.3 Measures

##### Baseline patient characteristics

We assessed baseline patient characteristics, including the diagnosis period (2008–2010, 2011–2014, 2015–2019), age at diagnosis (66–69, 70–74, 75–79, 80–84, 85+), sex (male, female), race and ethnicity (non‐Hispanic [NH] White, NH Other [NH Black, NH Asian/Pacific Islander, NH American Indian/Alaska Native], NH Unknown Race, Hispanic), the NCI Comorbidity Index^14^ measured during the 12‐month prediagnosis period (0, >0 to ≤1, >1), surgery status per SEER (yes [resection, tumor destruction, surgery not otherwise specified], no), which served as a proxy measure for whether patients had resectable or unresectable melanoma, stage at diagnosis (3, 4), marital status (single/widowed/divorced/separated, married/domestic partner), low‐income subsidy enrollment status (none, at least 1 month of enrollment during the 12‐month prediagnosis period), county‐level median household income (<$55,000, $55,000–$64,999, $65,000–$74,999, >$75,000), and US geographic region (Midwest, Northeast, South, West). Derived AJCC stage 6th edition was used to classify stage for diagnosis years 2008 and 2009, derived AJCC stage 7th edition was used for 2010–2015, derived SEER combined stage (2016–2017) was used for 2016 and 2017, and derived EOD 2018 stage group was used for 2018 and 2019.

##### Systemic treatment

We assessed any melanoma‐directed systemic treatment receipt from the date of diagnosis until death, or 12 months (including the month of diagnosis), whichever occurred first, and the first systemic treatment regimen administered or dispensed following diagnosis. The date of diagnosis was set to the first of the month of diagnosis as only the month and year of diagnosis are available in the data. We identified systemic treatments from the CoRe systemic AB and D files. Generic drug names were used to identify treatments (Table S1). We also identified “unspecified” drugs, i.e., claims with an ICD‐9/10 diagnosis/procedure code or HCPCS code indicating cancer systemic treatment administration but without a drug‐specific HCPCS or NDC code for the same claim ID and date combination. The first systemic treatment regimen was defined as the first systemic agent(s) beginning on or after the date of diagnosis, and any other agents started within 28 days of the first systemic agent. Treatment regimens were categorized as: (a) BRAF inhibitor with or without a MEK inhibitor, (b) ipilimumab (CTLA‐4) monotherapy, (c) PD‐1 inhibitor monotherapy (which included pembrolizumab or nivolumab), (d) ipilimumab plus PD‐1 inhibitor combination therapy, (e) cytokines, cytotoxic chemotherapy agents (e.g., interferon alfa‐2b, dacarbazine, temozolomide) and/or other melanoma systemic agents (e.g., tamoxifen), (f) ICI with any other agent(s), i.e., ICI plus a BRAF/MEK inhibitor, cytokines, cytotoxic chemotherapy or other agent, and (g) unspecified drug(s), i.e., ICD‐9/10 diagnosis/procedure code or HCPCS code indicating cancer systemic treatment administration but without a drug‐specific code (and no other drug‐specific code found within the 28‐day window).

#### 2.1.4 Analysis

##### Cohort characteristics & trends in any systemic treatment utilization

We first examined the sociodemographic and clinical characteristics of all patients included in the analysis. Characteristics of patients who received any systemic treatment during the first 12 months of diagnosis were described using frequencies and percentages, stratified by stage. We assessed the number and proportion of patients who received any systemic melanoma treatment during each diagnosis period (2008–2010, 2011–2014, 2015–2019), stratified by stage. In addition, we described the characteristics of patients who received any systemic treatment during the first 12 months of diagnosis, stratified by stage and diagnosis period, to gain further insight on the characteristics of patients receiving systemic treatment in each period.

##### Factors associated with any melanoma systemic treatment utilization

Multivariable logistic regression was used to estimate covariate‐adjusted odds ratios (aORs) and 95% confidence intervals (CIs) to identify factors associated with any systemic treatment receipt among patients with stage 3 and 4 melanoma separately. All baseline patient characteristics were included in the regression model except for race and ethnicity, which could not be included due to the small number of patients in the NH Other, NH Unknown, and Hispanic categories. Variables were selected a priori.

##### Trends in first systemic treatment type

Among patients who received systemic treatment, the type of first systemic treatment was described as frequencies and proportions, stratified by stage and diagnosis period (2008–2010, 2011–2014, 2015–2019). These periods were chosen to capture systemic treatment utilization: (1) prior to the introduction of ICIs and BRAF/MEK inhibitors (2008–2010), (2) following the approval of the first ICI (ipilimumab; CTLA‐4 inhibitor) and BRAF inhibitor (2011–2014), and (3) following the approval of PD‐1 inhibitors (2015–2019). In addition, we described the characteristics of patients with stage 4 melanoma who received PD‐1 inhibitor monotherapy versus ipilimumab and PD‐1 inhibitor combination therapy.

### 2.2 Survival analysis

We used the November 2022 submission of the SEER‐17 registries data to assess for changes in survival for patients diagnosed with stage 3 and 4 malignant melanoma, and aged <66 years and ≥ 66 years old. The survival analysis was not limited to patients in SEER‐Medicare. Patients with tumors diagnosed through death certificate/autopsy only, patients with missing/unknown cause of death, and patients lost to follow‐up in the month of diagnosis (alive with no survival time) were excluded from the analysis. The analysis was limited to patients with first primary tumors only (sequence number 0 or 1). Using the SEER*Stat software,^15^ we calculated up to 5‐year cause‐specific survival. Note that 1‐year cause‐specific survival was calculated for patients diagnosed between 2004 and 2019, and 5‐year cause‐specific survival for patients diagnosed between 2004 and 2015 as we had follow‐up data only through 2020.

We then used the JPSurv webtool^16^, ^17^ to characterize cause‐specific survival trends by year of diagnosis. JPSurv estimates the location and number of Joinpoints, i.e., years at diagnosis when changes in survival trends occurred, and the average absolute change (AAC) in survival between Joinpoints to understand how much survival has been increasing or decreasing. The year of diagnosis range was set to 2004–2019, and a maximum of two Joinpoints were tested. All other settings were set to default. The final model for each age and stage combination was selected based on the Bayesian Information Criterion and Akaike Information Criterion. This study did not require Institutional Review Board review as secondary analyses of de‐identified data are not considered human subjects research by the National Institutes of Health.

## 3 RESULTS

### 3.1 Cohort characteristics & trends in any systemic treatment utilization

**FIGURE 2:**
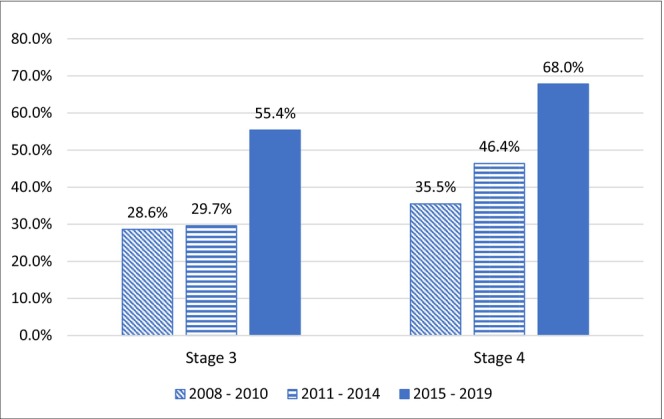
Proportion of patients with stage 3 and 4 melanoma receiving any melanoma systemic treatment (2008–2010, 2011–2014, 2015–2019).

The final cohort included 2012 patients diagnosed with stage 3 and 4 melanoma between 2008 and 2019 (Table 1). Among the 1198 and 814 patients with stage 3 stage 4 melanoma, 43.2% and 56.8% received melanoma‐directed systemic therapy in the first year following diagnosis, respectively. Overall, there was an almost two‐fold increase in the use of systemic treatment over time, from 28.6% (2008–2010) to 55.4% (2015–2019) among patients with stage 3 melanoma, and from 35.5% (2008–2010) to 68.0% (2015–2019) among patients with stage 4 melanoma (Figure 2). Table S2 describes the characteristics of patients who received any systemic treatment during the first 12 months of diagnosis, stratified by stage and diagnosis period.

**TABLE 1:** Characteristics of patients diagnosed with stage 3/4 melanoma in 2008–2019 and receipt of any systemic treatment within 12 months of diagnosis, by stage, SEER‐Medicare CoRe.

Patient characteristic		Stage 3 (*n* = 1198)		Stage 4 (*n* = 814)	
	Overall	Stage 3 total	Systemic treatment received^a^	Stage 4 total	Systemic treatment received^a^
	*n* (col %)	*n* (col %)	*n* (row %)	*n* (col %)	*n* (row %)
Total	**2012**	1198 (100.0%)	517 (43.2%)	814 (100.0%)	462 (56.8%)
Age at diagnosis, years					
66–69	438 (21.8%)	282 (23.5%)	131 (46.5%)	156 (19.2%)	102 (65.4%)
70–74	508 (25.3%)	309 (25.8%)	156 (50.5%)	199 (24.5%)	127 (63.8%)
75–79	439 (21.8%)	260 (21.7%)	111 (42.7%)	179 (22.0%)	105 (58.7%)
80–84	323 (16.1%)	189 (15.8%)	68 (36.0%)	134 (16.5%)	76 (56.7%)
85+	304 (15.1%)	158 (13.2%)	51 (32.3%)	146 (17.9%)	52 (35.6%)
Sex					
Male	1235 (61.4%)	714 (59.6%)	325 (45.5%)	521 (64.0%)	310 (59.5%)
Female	777 (38.6%)	484 (40.4%)	192 (39.7%)	293 (36.0%)	152 (51.9%)
Race/ethnicity					
NH White	1876 (93.2%)	1107 (92.4%)	474 (42.8%)	769 (94.5%)	441 (57.4%)
NH Other					
NH Black	17 (0.8%)	<11 (<0.9%)^b^	<11^b^	<11 (<1.4%)^b^	<11^b^
NH Asian/PI	21 (1.0%)	12 (1.0%)	<11^b^	<11 (<1.4%)^b^	<11^b^
NH American Indian/Alaska Native	<11 (<0.5%)^b^	<11 (<0.9%)^b^	<11^b^	<11 (<1.4%)^b^	<11^b^
NH unknown race	<11 (<0.5%)^b^	<11 (<0.9%)^b^	<11^b^	<11 (<1.4%)^b^	<11^b^
Hispanic (Any race)	87 (4.3%)	64 (5.3%)	30 (46.9%)	23 (2.8%)	<11 (<47.8%)^b^
NCI Comorbidity Index					
0	855 (42.5%)	559 (46.7%)	250 (44.7%)	296 (36.4%)	186 (62.8%)
>0 to ≤1	841 (41.8%)	470 (39.2%)	206 (43.8%)	371 (45.6%)	214 (57.7%)
>1	316 (15.7%)	169 (14.1%)	61 (36.1%)	147 (18.1%)	62 (42.2%)
Diagnosis period					
2008–2010	306 (15.2%)	182 (15.2%)	52 (28.6%)	124 (15.2%)	44 (35.5%)
2011–2014	618 (30.7%)	381 (31.8%)	113 (29.7%)	237 (29.1%)	110 (46.4%)
2015–2019	1088 (54.1%)	635 (53.0%)	352 (55.4%)	453 (55.7%)	308 (68.0%)
Surgery					
No	762 (37.9%)	154 (12.9%)	75 (48.7%)	608 (74.7%)	346 (56.9%)
Yes	1250 (62.1%)	1044 (87.2%)	442 (42.3%)	206 (25.3%)	116 (56.3%)
Marital status					
Single/widowed	744 (37.0%)	417 (34.8%)	163 (39.1%)	327 (40.2%)	151 (46.2%)
Married/domestic partner	1268 (63.0%)	781 (65.2%)	354 (45.3%)	487 (59.8%)	311 (63.9%)
Low‐income subsidy enrollment					
No	1657 (82.4%)	1009 (84.2%)	454 (45.0%)	648 (79.6%)	392 (60.5%)
At least 1 month	355 (17.6%)	189 (15.8%)	63 (33.3%)	166 (20.4%)	70 (42.2%)
County‐level median household income ($)					
<55,000	556 (27.6%)	330 (27.6%)	130 (39.4%)	226 (27.8%)	123 (54.4%)
55,000–64,999	406 (20.2%)	254 (21.2%)	102 (40.2%)	152 (18.7%)	76 (50.0%)
65,000–74,999	419 (20.8%)	252 (21.0%)	118 (46.8%)	167 (20.5%)	100 (59.9%)
>75,000	631 (31.4%)	362 (30.2%)	167 (46.1%)	269 (33.1%)	163 (60.6%)
Geographic region					
Midwest	216 (10.7%)	142 (11.9%)	56 (39.4%)	74 (9.1%)	37 (50.0%)
Northeast	363 (18.0%)	203 (16.9%)	75 (37.0%)	160 (19.7%)	92 (57.5%)
South	492 (24.5%)	292 (24.4%)	128 (43.8%)	200 (24.6%)	102 (51.0%)
West	941 (46.8%)	561 (46.8%)	258 (46.0%)	380 (46.7%)	231 (60.8%)

^a^ Systemic therapy includes any systemic agent(s) listed in Supplemental Table S1 and any “unspecified” agent(s).

^b^ Per SEER‐Medicare confidentiality policies, cell sizes <11 have been masked.

### 3.2 Factors associated with any melanoma systemic treatment utilization

The multivariate logistic regression (Table 2) assessing factors associated with the receipt of any systemic treatment indicated that the odds of receiving systemic treatment were significantly higher for patients diagnosed in 2015–2019 than 2008–2010 (stage 3: aOR 3.01 [95% CI: 2.09–4.34]; stage 4: aOR 4.00 [95% CI: 2.55–6.28]). The oldest age group had lower odds of receiving systemic treatment than the youngest age group (stage 3: aOR 0.58 [95% CI: 0.38–0.89]; stage 4: aOR 0.29 [95% CI: 0.17–0.49]). Among patients with stage 4 melanoma, being married or having a domestic partner was associated with higher odds of systemic treatment receipt, and an NCI comorbidity index of >1 was associated with lower odds of treatment receipt compared to having an index of zero. Among patients with stage 3 melanoma, LIS enrollment status was associated with lower odds of systemic treatment receipt.

**TABLE 2:** Association between patient characteristics and receipt of any systemic treatment within 12 months of diagnosis among patients diagnosed with stage 3/4 melanoma, by stage, SEER‐Medicare CoRe.

Patient characteristic	Stage 3 (*n* = 1198)		Stage 4 (*n* = 814)	
	Adjusted OR	95% CI	Adjusted OR	95% CI
Age at diagnosis, years				
66–69	ref		ref	
70–74	1.05	0.75–1.48	0.83	0.52–1.34
75–79	0.77	0.54–1.11	0.71	0.44–1.16
80–84	0.60	0.40–0.90	0.63	0.38–1.06
85+	0.58	0.38–0.89	0.29	0.17–0.49
Sex				
Male	ref		ref	
Female	0.87	0.67–1.12	1.01	0.72–1.43
NCI Comorbidity Index				
0	ref		ref	
>0 to ≤1	0.99	0.76–1.29	0.84	0.60–1.19
>1	0.75	0.51–1.11	0.47	0.30–0.74
Diagnosis period				
2008–2010	ref		ref	
2011–2014	1.02	0.69–1.52	1.63	1.01–2.63
2015–2019	3.01	2.09–4.34	4.00	2.55–6.28
Surgery				
No	ref		ref	
Yes	0.74	0.52–1.06	1.10	0.77–1.57
Marital status				
Single/widowed	ref		ref	
Married/domestic partner	1.03	0.78–1.36	1.68	1.19–2.37
Low‐income subsidy enrollment				
No	ref		ref	
At least 1 month	0.69	0.48–0.99	0.78	0.52–1.19
County‐level median household income ($)				
<55,000	ref		ref	
55,000‐64,999	1.05	0.73–1.51	0.77	0.48–1.25
65,000‐74,999	1.33	0.90–1.97	0.97	0.58–1.62
>75,000	1.29	0.88–1.89	0.97	0.59–1.60
Geographic region				
Midwest	ref		ref	
Northeast	0.89	0.54–1.47	1.53	0.79–2.97
South	1.41	0.91–2.19	1.10	0.60–2.02
West	1.32	0.88–1.99	1.51	0.86–2.66

### 3.3 Trends in first systemic treatment type

There were substantial changes in melanoma systemic treatment utilization over the study period. Among patients diagnosed in 2008–2010 receiving systemic treatment, the standard was cytokines, cytotoxic chemotherapy, and/or other agents, e.g., tamoxifen. However, among patients diagnosed in 2015–2019, a small percent received these therapies (stage 3: 5.1%, stage 4: <3.6%; Table 3), with the dominant treatment being PD‐1 inhibitors, which was used as monotherapy by 69.9% of patients with stage 3 melanoma and 54.6% of patients with stage 4 melanoma, and in combination with ipilimumab by an additional 4.0% of patients with stage 3 melanoma and 22.7% of patients with stage 4 melanoma. The use of BRAF/MEK inhibitors among patients diagnosed in 2011–2014 compared with 2015–2019 declined from 12.4% to 3.7% (stage 3) and from 15.5% to 8.4% (stage 4). Characteristics of patients with stage 4 melanoma who received PD‐1 inhibitor monotherapy versus ipilimumab and PD‐1 inhibitor combination therapy are described in Table S3.

**TABLE 3:** Type of first systemic treatment among patients with stage 3/4 melanoma who received any systemic treatment within 12 months of diagnosis (diagnosis years 2008–2010, 2011–2014, 2015–2019), by stage, SEER‐Medicare CoRe.

First systemic treatment regimen	Stage 3 (*n* = 517)			Stage 4 (*n* = 462)		
	2008–2010 (*n* = 52)	2011–2014 (*n* = 113)	2015–2019 (*n* = 352)	2008–2010 (*n* = 44)	2011–2014 (*n* = 110)	2015–2019 (*n* = 308)
Cytokines/cytotoxic chemotherapy/other	>41 (>78.8%)	42 (37.2%)	18 (5.1%)	>33 (>75.0%)	25 (22.7%)	<11 (<3.6%)^c^
BRAF +/− MEK inhibitor	0	14 (12.4%)	13 (3.7%)	0	17 (15.5%)	26 (8.4%)
Ipilimumab monotherapy	0	24 (21.2%)	27 (7.7%)	0	52 (47.3%)	13 (4.2%)
PD‐1 inhibitor monotherapy	0	<11 (<9.7%)^c^	246 (69.9%)	0	<11 (<10.0%)^c^	168 (54.6%)
Ipilimumab + PD‐1 inhibitor combination	0	0	14 (4.0%)	0	0	70 (22.7%)
Immune checkpoint Inhibitor^a^ + Any other^b^	0	<11 (<9.7%)^c^	0	0	<11 (<10.0%)^c^	<11 (<3.6%)^c^
Unspecified drug(s)	<11 (<21.2%)^c^	28 (24.8%)	34 (9.7%)	<11 (<25.0%)^c^	13 (11.8%)	20 (6.5%)

^a^ Immune Checkpoint Inhibitor includes ipilimumab and/or PD‐1 inhibitor.

^b^ “Any Other” includes BRAF/MEK inhibitor(s)/Cytokines/Cytotoxic chemotherapy/Other agents.

^c^ Per SEER‐Medicare confidentiality policies, cell sizes <11 have been masked.

### 3.4 Survival

**FIGURE 3:**
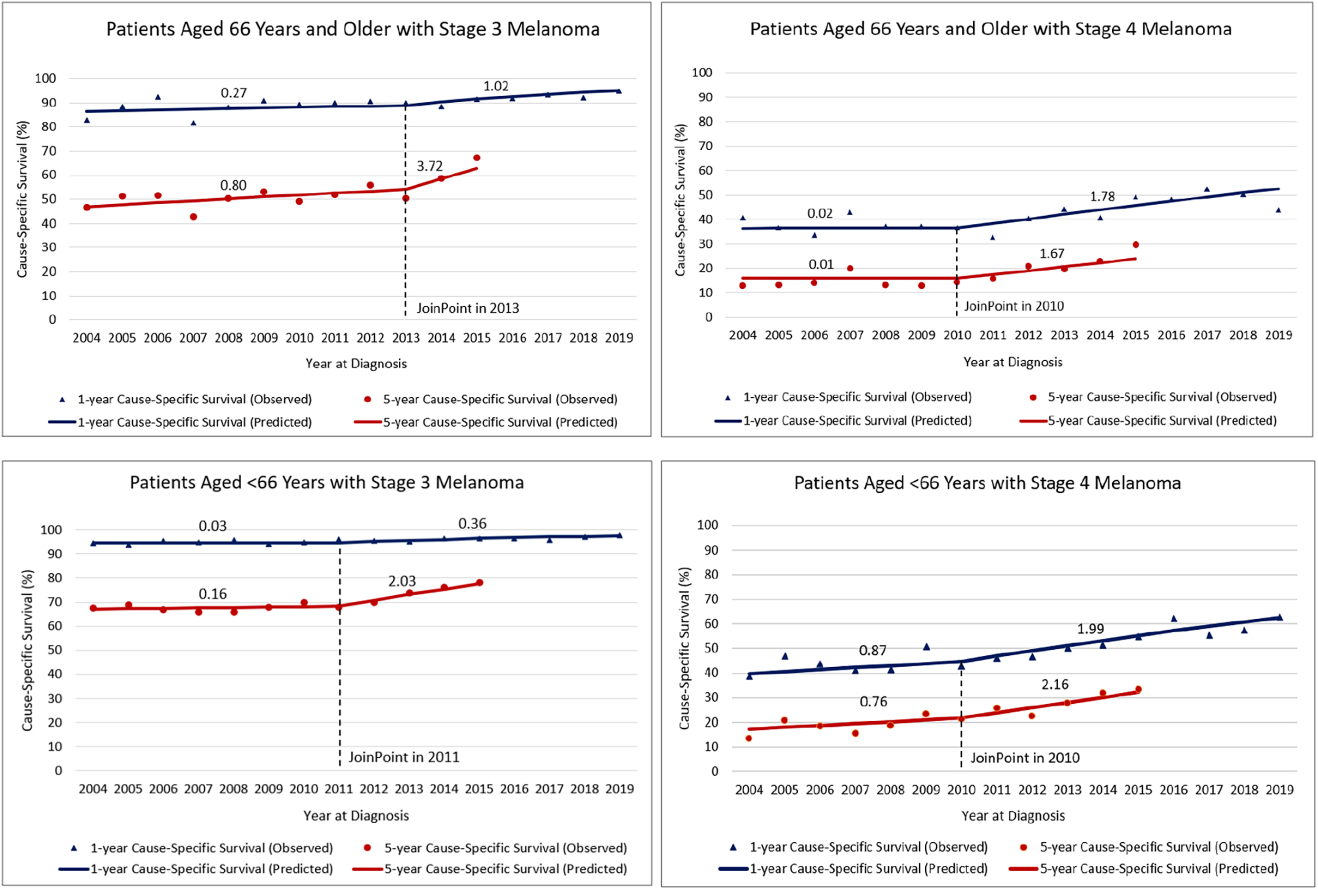
1‐year and 5‐year cause‐specific survival by year of diagnosis. Figures shown by age (≥66 vs. <66 years) and stage (stage 3 vs. 4).

For stage 4 melanoma, Joinpoints were estimated in 2010 for both age groups (<66 and 66+) (Figure 3). Conversely, for stage 3, changes in survival trends were estimated in 2011 (ages <66) and in 2013 (ages 66+). Post‐Joinpoints, all groups—stage 3 and 4, and age groups <66 and 66 + —showed a steeper increase in both 1‐year and 5‐year survival compared to the prior period. In some cases, survival was not increasing in the prior period (Table S4). The greatest improvement in 5‐year survival was observed for patients aged 66+ years with stage 3 melanoma (3.72% [95% CI: 2.81–4.62%]). Among patients with stage 3 melanoma, patients aged 66+ years demonstrated a greater improvement in 5‐year survival than patients aged <66 years (3.72% [95% CI: 2.81–4.62%] vs. 2.03% [95% CI: 1.62–2.44%]), and for stage 4 melanoma, improvements in survival were similar between older and younger patients (1.67% [95% CI: 1.14–2.21%] vs. 2.16% [95% CI: 1.67–2.65%]).

Although the 5‐year survival data is currently only available for patients diagnosed through 2015, the models include 1‐year survival rates for patients diagnosed between 2016 and 2019 and the results indicate a continuing improvement in survival.

## 4 DISCUSSION

This study examined temporal trends in the utilization of systemic therapies and changes in survival trends among older patients with advanced melanoma. The results of our study demonstrated that the utilization of systemic therapies in this population almost doubled from 2008 to 2019, from 28.6% to 55.4% among patients with stage 3 melanoma, and from 35.5% to 68.0% among patients with stage 4 melanoma. The increased use of systemic therapies over time is likely due to the introduction of PD‐1 inhibitors which are more effective and less toxic than the traditional cytotoxic chemotherapy agents and cytokines which were the standard of care prior to 2011.

The increased use of systemic therapies and the shift in the melanoma treatment landscape align with the improvements in cause‐specific survival among older patients with melanoma. Whereas before 2010, survival was not increasing, both 1‐year and 5‐year survival have been increasing since 2010 among patients aged 66+ years with stage 4 melanoma. Among patients aged 66+ years with stage 3 melanoma, increases in survival trends were observed beginning a few years later in 2013, which may be due to slower adoption of ICIs and BRAF/MEK inhibitors. Among patients with stage 3 melanoma, older individuals had greater improvements in survival than younger individuals, and for stage 4 melanoma, improvements in survival were similar between older and younger patients. These findings indicate that improvements in survival in the new era of melanoma treatment are similar, if not greater, among older patients with advanced melanoma compared with younger patients, and appear to be continuing to improve over time.

In our study, patients aged 85+ years had lower odds of systemic treatment receipt compared with the youngest age group (66–69 years). Older patients may be more frail and there may be greater concerns about treatment‐related morbidity.^18^ It is possible that the perceived risks outweigh the perceived benefits of systemic treatment for some older patients, or systemic treatment might not be part of the patient's goals of care. Among patients with stage 4 melanoma, a higher comorbidity burden was also associated with lower odds of receiving systemic treatment, similar to the findings of other cancer studies.^19^, ^20^, ^21^ The observed association between comorbidity and treatment utilization may be related to concerns for treatment tolerability among patients with a higher comorbidity burden. Interestingly, patients with stage 4 melanoma who were married or had a domestic partner were more likely to receive systemic treatment than patients who were single or widowed. This result agrees with findings from previous cancer literature.^11^, ^22^, ^23^

Our analysis revealed a clear shift in the treatment landscape between 2008 and 2019, with an uptake of ICIs as first‐line therapy, and a decline in the use of traditional cytotoxic chemotherapy agents and cytokines. The use of ipilimumab in combination with a PD‐1 inhibitor was lower overall than the use of PD‐1 inhibitor monotherapy, and this is likely due to increased concerns about the toxicities of combination treatment.^24^ The results showed that ipilimumab plus PD‐1 inhibitor combination therapy was higher among patients with stage 4 melanoma compared with patients with stage 3 melanoma. This is not surprising as ICI combination treatment is approved for unresectable or metastatic melanoma and is more likely to be used among patients with more severe disease, such as patients with brain metastases. Additionally, our results indicate that BRAF/MEK inhibitor use has not been as high as the ICIs. This agrees with the findings of the study by Lee et al. which also saw lower use of BRAF/MEK inhibitors compared with ICIs, and a decline in the use of BRAF/MEK inhibitors after 2015 among a cohort of older patients diagnosed with advanced melanoma between 2012 and 2017 who received immunotherapy or targeted therapy.^7^ Use of BRAF/MEK inhibitors may be lower than the use of ICIs because BRAF/MEK inhibitors are only indicated for patients with BRAF mutations whereas ICIs can be used regardless of patients' mutation status. In the DREAMseq study,^25^ first‐line combination immunotherapy was found to be superior to first‐line targeted therapy (dual BRAF/MEK inhibition) in patients with BRAF mutations. Although this study was published after the observation period that we employed, advanced knowledge of some results presented at an American Society of Clinical Oncology (ASCO) virtual plenary session could have contributed to higher utilization of ICIs than BRAF/MEK inhibitors. In addition, the need for molecular testing for mutations adds another step and may influence treatment selection, especially outside of academic medical centers. A molecular testing rate of 73.8% has been observed in a study by Hill et al,^25^ showing that not all patients receive molecular testing.

The findings of this study need to be considered in the context of its limitations. Treatments provided through clinical trials are not captured in the Medicare claims, and hence we may have underestimated the number of patients receiving systemic treatment within our study population. However, the number of patients in this 66+ Medicare population participating in clinical trials is likely very few.^26^, ^27^, ^28^

It should be noted that most patients with metastatic melanoma are initially diagnosed with early‐stage disease and thus may not be treated with systemic therapy unless they have a recurrence. We were not able to assess systemic therapy among patients with recurrent disease because neither SEER nor Medicare data reliably identify recurrence. Thus, our study represents the subgroup presenting with advanced disease, which may differ from those who develop unresectable or metastatic disease later. Also, some patients with stage 3 melanoma at diagnosis may have had rapid disease progression to metastatic melanoma by the time of initiating systemic treatment, but we were unable to identify and address disease progression using the current dataset.

We did not have BRAF or KIT mutation status; therefore, we could not assess whether treatments were prescribed by mutation status. Resectability is a key factor mentioned in multiple FDA drug labels for melanoma indications but is not a concept that is captured by cancer registries. We used surgery receipt as a proxy for distinguishing between resectable and unresectable melanoma; however, there may have been patients with resectable melanoma who did not receive surgery, especially patients with greater comorbidity burden or poor performance status.

We did not limit the survival analysis to patients in the treatment utilization analysis, as the small sample size would not allow us to examine trends. Instead, we used SEER data for the survival analysis which also allowed for estimation of survival trends for patients younger than 66 years. In addition, it is possible that someone diagnosed in one period switched to a new drug approved in the next period, which may have lengthened their survival. Since we did not examine these later switches, we may be underestimating the role of new treatments on survival.

Despite these limitations, this study has important strengths. This study adds to the literature with its use of recent data that include patients diagnosed between 2008 and 2019 and their claims through 2020, as well as updated survival estimates. Also, the study demonstrates the use of SEER‐Medicare CoRe, a newly developed dataset designed to facilitate the use of SEER‐Medicare data with more ease.

## 5 CONCLUSIONS

This study shows a significant increase in the utilization of systemic therapy among older patients with de novo advanced melanoma, and a rapid uptake of highly effective systemic agents, notably ICIs, in a deadly disease that had few effective treatment options before 2011. The substantial uptake of ICIs as well as increased use of systemic treatment overall align with the improvement in survival observed among patients with advanced melanoma.

## AUTHOR CONTRIBUTIONS

**Yoon Duk Hong:** Conceptualization (lead); formal analysis (lead); methodology (lead); project administration (lead); visualization (lead); writing – original draft (lead); writing – review and editing (equal). **Lindsey Enewold:** Conceptualization (equal); data curation (lead); methodology (equal); resources (lead); supervision (lead); writing – review and editing (equal). **Elad Sharon:** Conceptualization (equal); methodology (equal); writing – review and editing (equal). **Jeremy L. Warner:** Conceptualization (equal); methodology (equal); writing – review and editing (equal). **Amy J. Davidoff:** Conceptualization (equal); methodology (equal); writing – review and editing (equal). **Chris Zeruto:** Data curation (lead); validation (lead); writing – review and editing (equal). **Angela B. Mariotto:** Conceptualization (equal); data curation (lead); methodology (equal); resources (lead); supervision (lead); writing – review and editing (equal).

## FUNDING INFORMATION

This work was supported by the National Cancer Institute at the National Institutes of Health.

## CONFLICT OF INTEREST STATEMENT

YDH, LE, AJD, CZ and ABM have nothing to report. JLW reports: Research funding – NIH, AACR, Brown Physicians Incorporated; Consulting – Westat, The Lewin Group; Editorial – ASCO. ES reports: Advisory board – Mallinckrodt Pharmaceuticals*; Consulting – D.E. Shaw Research*.

*Subsequent to employment at the NCI, during which time ES contributed to this manuscript.

## Supporting information


Table S1.

## Data Availability

SEER‐Medicare data may be requested by submitting an application: https://healthcaredelivery.cancer.gov/seermedicare/obtain/requests.html. Access to the CoRe files is granted through the SEER‐Medicare application process.
