# Supplementary material for: Evolving patterns in systemic treatment utilization and survival among older patients with advanced cutaneous melanoma
Source: Cancer Med. 2024 Aug 28;13(16):e70131. doi: 10.1002/cam4.70131 (PMC11350836; doi:10.1002/cam4.70131)
Supplement: Supplementary file 1 — Table S1. [file CAM4-13-e70131-s001.docx]

**Supplemental Table 1**. Melanoma systemic treatment categorization

| **Drug Name** | **Treatment Category** |
| --- | --- |
| **BRAF Inhibitor** | BRAF +/- MEK inhibitor |
| DABRAFENIB |  |
| ENCORAFENIB |  |
| VEMURAFENIB |  |
| **MEK Inhibitor** |  |
| BINIMETINIB |  |
| COBIMETINIB |  |
| TRAMETINIB |  |
| **CTLA-4 Inhibitor** | Ipilimumab monotherapy |
| IPILIMUMAB |  |
| **PD-1 Inhibitor** | PD-1 inhibitor monotherapy |
| NIVOLUMAB |  |
| PEMBROLIZUMAB |  |
| IPILIMUMAB + NIVOLUMAB or PEMBROLIZUMAB | Ipilimumab + PD-1 inhibitor combination |
| **PD-L1 Inhibitor** | PD-L1 inhibitor |
| ATEZOLIZUMAB |  |
| **Cytokines** | Cytokines/Cytotoxic chemotherapy/Other |
| INTERFERON ALFA-2A |  |
| INTERFERON ALFA-2B |  |
| PEGINTERFERON ALFA-2A |  |
| PEGINTERFERON ALFA-2B |  |
| ALDESLEUKIN |  |
| INTERFERON GAMMA-1B |  |
| **Cytotoxic Chemotherapy** |  |
| CARBOPLATIN |  |
| PACLITAXEL/PACLITAXEL, PROTEIN BOUND |  |
| TEMOZOLOMIDE |  |
| DACARBAZINE |  |
| CISPLATIN |  |
| CARMUSTINE |  |
| DOCETAXEL |  |
| VINBLASTINE |  |
| VINCRISTINE/VINCRISTINE SULFATE |  |
| HYDROXYUREA |  |
| **Other Drugs** |  |
| TAMOXIFEN/TAMOXIFEN CITRATE |  |
| IMATINIB/ IMATINIB MESYLATE |  |
| SUNITINIB MALATE |  |
| BORTEZOMIB |  |
| BEVACIZUMAB |  |
| SORAFENIB |  |
| SARGRAMOSTIM |  |
| BACILLUS CALMETTE-GUERIN / BACILLUS CALMETTE-GUERIN SUBSTRAIN NICE LIVE ANTIGEN / BCG |  |

Note: Atezolizumab (PDL-1 inhibitor), peginterferon alfa-2a, interferon gamma-1b, vincristine, and sargramostim were searched for, but no records were identified in the claims records of patients included in this study.

BRAF = v-raf murine sarcoma viral oncogene homolog B1, MEK = mitogen-activated protein kinase kinase, CTLA-4 = cytotoxic T-lymphocyte–associated antigen 4, PD-1 = Programmed death-1, PD-L1 = programmed death ligand-1.

**Supplemental Table 2**. Characteristics of patients with stage 3/4 melanoma receiving any systemic treatment within 12 months of diagnosis, by stage and diagnosis period (2008-2010, 2011-2014, 2015-2019), SEER-Medicare CoRe.

|  | Stage 3 (n = 1,198) | | | | | | Stage 4 (n = 814) | | | | | |
| --- | --- | --- | --- | --- | --- | --- | --- | --- | --- | --- | --- | --- |
| Patient Characteristic | **2008 – 2010** | | **2011 – 2014** | | **2015 – 2019** | | **2008 – 2010** | | **2011 – 2014** | | **2015 – 2019** | |
|  | **Total** | **Systemic Treatment Received** *^a^* | **Total** | **Systemic Treatment Received** *^a^* | **Total** | **Systemic Treatment Received** *^a^* | **Total** | **Systemic Treatment Received** *^a^* | **Total** | **Systemic Treatment Received** *^a^* | **Total** | **Systemic Treatment Received** *^a^* |
|  | n  (col %) | n  (row %) | n  (col %) | n  (row %) | n  (col %) | n  (row %) | n  (col %) | n  (row %) | n  (col %) | n  (row %) | n  (col %) | n  (row %) |
| Total | 182  (100%) | 52  (28.6%) | 381  (100%) | 113  (29.7%) | 635  (100%) | 352  (55.4%) | 124  (100%) | 44  (35.5%) | 237  (100%) | 110  (46.4%) | 453  (100%) | 308  (68.0%) |
| Age at Diagnosis, years |  |  |  |  |  |  |  |  |  |  |  |  |
| 66-69 | 48  (26.4%) | 18  (37.5%) | 98  (25.7%) | 36  (36.7%) | 136  (21.4%) | 77  (56.6%) | 27  (21.8%) | <11*^a^* | 44  (18.6%) | 29  (65.9%) | 85  (18.8%) | 63  (74.1%) |
| 70-74 | 35  (19.2%) | 13  (37.1%) | 99  (26.0%) | 34  (34.3%) | 175  (27.6%) | 109  (62.3%) | 30  (24.2%) | 12  (40.0%) | 59  (24.9%) | 37  (62.7%) | 110  (24.3%) | 78  (70.9%) |
| 75-79 | 42  (23.1%) | >11 | 66  (17.3%) | 16  (24.2%) | 152  (23.9%) | 80  (52.6%) | 30  (24.2%) | 13  (43.3%) | 47  (19.8%) | 18  (38.3%) | 102  (22.5%) | 74  (72.6%) |
| 80-84 | 35  (19.2%) | <11*^a^* | 56  (14.7%) | 12  (21.4%) | 98  (15.4%) | 51  (52.0%) | 16  (12.9%) | <11*^a^* | 36  (15.2%) | >11 | 82  (18.1%) | 55  (67.1%) |
| 85+ | 22  (12.1%) | <11*^a^* | 62  (16.3%) | 15  (24.2%) | 74  (11.7%) | 35  (47.3%) | 21  (16.9%) | <11*^a^* | 51  (21.5%) | <11*^a^* | 74  (16.3%) | 38  (51.4%) |
| Sex |  |  |  |  |  |  |  |  |  |  |  |  |
| Male | 97  (53.3%) | 34  (35.1%) | 233  (61.2%) | 72  (30.9%) | 384  (60.5%) | 219  (57.0%) | 72  (58.1%) | 26  (36.1%) | 148  (62.5%) | 73  (49.3%) | 301  (66.5%) | 211  (70.1%) |
| Female | 85  (46.7%) | 18  (21.2%) | 148  (38.9%) | 41  (27.7%) | 251  (39.5%) | 133  (53.0%) | 52  (41.9%) | 18  (34.6%) | 89  (37.6%) | 37  (41.6%) | 152  (33.6%) | 97  (63.8%) |
| NCI Comorbidity Index |  |  |  |  |  |  |  |  |  |  |  |  |
| 0 | 83  (45.6%) | 27  (32.5%) | 188  (49.3%) | 63  (33.5%) | 288  (45.4%) | 160  (55.6%) | 49  (39.5%) | 19  (38.8%) | 89  (37.6%) | 56  (62.9%) | 158  (34.9%) | 111  (70.3%) |
| >0 to <=1 | 72  (39.6%) | >11 | 143  (37.5%) | 36  (25.2%) | 255  (40.2%) | 151  (59.2%) | 55  (44.4%) | >11 | 107  (45.2%) | >11 | 209  (46.1%) | 149  (71.3%) |
| >1 | 27  (14.8%) | <11*^a^* | 50  (13.1%) | 14  (28.0%) | 92  (14.5%) | 41  (44.6%) | 20  (16.1%) | <11*^a^* | 41  (17.3%) | <11*^a^* | 86  (19.0%) | 48  (55.8%) |
| Surgery |  |  |  |  |  |  |  |  |  |  |  |  |
| No | 20  (11.0%) | <11*^a^* | 54  (14.2%) | 20  (37.0%) | 80  (12.6%) | 49  (61.3%) | 90  (72.6%) | 33  (36.7%) | 177  (74.7%) | 85  (48.0%) | 341  (75.3%) | 228  (66.9%) |
| Yes | 162  (89.0%) | >11 | 327  (85.8%) | 93  (28.4%) | 555  (87.4%) | 303  (54.6%) | 34  (27.4%) | 11  (32.4%) | 60  (25.3%) | 25  (41.7%) | 112  (24.7%) | 80  (71.4%) |
| Marital Status |  |  |  |  |  |  |  |  |  |  |  |  |
| Single/widowed | 76  (41.8%) | 18  (23.7%) | 132  (34.7%) | 38  (28.8%) | 209  (32.9%) | 107  (51.2%) | 58  (46.8%) | 17  (29.3%) | 94  (39.7%) | 34  (36.2%) | 175  (38.6%) | 100  (57.1%) |
| Married/domestic partner | 106  (58.2%) | 34  (32.1%) | 249  (65.4%) | 75  (30.1%) | 426  (67.1%) | 245  (57.5%) | 66  (53.2%) | 27  (40.9%) | 143  (60.3%) | 76  (53.2%) | 278  (61.4%) | 208  (74.8%) |
| Low-Income Subsidy Enrollment |  |  |  |  |  |  |  |  |  |  |  |  |
| No | 152  (83.5%) | >11 | 312  (81.9%) | 92  (29.5%) | 545  (85.8%) | 313  (57.4%) | 76  (61.3%) | 27  (35.5%) | 184  (77.6%) | 94  (51.1%) | 388  (85.7%) | 271  (69.9%) |
| At Least 1 Month | 30  (16.5%) | <11*^a^* | 69  (18.1%) | 21  (30.4%) | 90  (14.2%) | 39  (43.3%) | 48  (38.7%) | 17  (35.4%) | 53  (22.4%) | 16  (30.2%) | 65  (14.4%) | 37  (56.9%) |
| County-level Median Household Income ($) |  |  |  |  |  |  |  |  |  |  |  |  |
| <55,000 | 55  (30.2%) | 18  (32.7%) | 127  (33.3%) | 35  (27.6%) | 148  (23.3%) | 77  (52.0%) | 38  (30.7%) | 15  (39.5%) | 73  (30.8%) | 33  (45.2%) | 115  (25.4%) | 75  (65.2%) |
| 55,000 – 64,999 | 44  (24.2%) | <11*^a^* | 85  (22.3%) | 26  (30.6%) | 125  (19.7%) | 68  (54.4%) | 25  (20.2%) | <11*^a^* | 59  (24.9%) | 31  (52.5%) | 68  (15.0%) | 40  (58.8%) |
| 65,000 – 74,999 | 40  (22.0%) | >11 | 70  (18.4%) | 20  (28.6%) | 142  (22.4%) | 86  (60.6%) | 25  (20.2%) | <11*^a^* | 35  (14.8%) | 17  (48.6%) | 107  (23.6%) | 75  (70.1%) |
| >75,000 | 43  (23.6%) | 14  (32.6%) | 99  (26.0%) | 32  (32.3%) | 220  (34.7%) | 121  (55.0%) | 36  (29.0%) | 16  (44.4%) | 70  (29.5%) | 29  (41.4%) | 163  (36.0%) | 118  (72.4%) |
| Geographic Region |  |  |  |  |  |  |  |  |  |  |  |  |
| Midwest | 23  (12.6%) | <11*^a^* | 36  (9.5%) | <11*^a^* | 83  (13.1%) | 42  (50.6%) | 11  (8.9%) | <11^a^ | 21  (8.9%) | 11  (52.4%) | 42  (9.3%) | 24  (57.1%) |
| Northeast | 28  (15.4%) | <11*^a^* | 73  (19.2%) | >11 | 102  (16.1%) | 51  (50.0%) | 25  (20.2%) | >11 | 41  (17.3%) | 18  (43.9%) | 94  (20.8%) | 62  (66.0%) |
| South | 42  (23.1%) | 12  (28.6%) | 105  (27.6%) | 32  (30.5%) | 145  (22.8%) | 84  (57.9%) | 38  (30.7%) | 13  (34.2%) | 64  (27.0%) | 22  (34.4%) | 98  (21.6%) | 67  (68.4%) |
| West | 89  (48.9%) | 28  (31.5%) | 167  (43.8%) | 55  (32.9%) | 305  (48.0%) | 175  (57.4%) | 50  (40.3%) | 17  (34.0%) | 111  (46.8%) | 59  (53.2%) | 219  (48.3%) | 155  (70.8%) |

*^a^* Per SEER-Medicare confidentiality policies, cell sizes <11 have been masked.

Note: race and ethnicity are not included in this table due to the majority being NH White.

**Supplemental Table 3**. Use of PD-1 inhibitor monotherapy versus ipilimumab-PD-1 inhibitor combination among patients with stage 4 melanoma, diagnosed 2015-2019, SEER-Medicare CoRe.

| Patient Characteristic | Stage 4 (n = 238) | |
| --- | --- | --- |
|  | **PD-1 inhibitor monotherapy** *^a^* | **Ipilimumab-PD-1 inhibitor combination** |
|  | **n (col %)** | **n (col %)** |
| Total | 168 (100%) | 70 (100%) |
| Age at Diagnosis, years |  |  |
| 66-69 | 27 (16.1%) | 24 (34.3%) |
| 70-74 | 36 (21.4%) | 23 (32.9%) |
| 75-79 | 46 (27.4%) | <11 (<15.7%) *^b^* |
| 80-84 | 32 (19.1%) | <11 (<15.7%) *^b^* |
| 85+ | 27 (16.1%) | <11 (<15.7%) *^b^* |
| Sex |  |  |
| Male | 113 (67.3%) | 49 (70.0%) |
| Female | 55 (32.7%) | 21 (30.0%) |
| NCI Comorbidity Index |  |  |
| 0 | 60 (35.7%) | 32 (45.7%) |
| >0 to <=1 | 76 (45.2%) | >27 (>38.6%) *^b^* |
| >1 | 32 (19.1%) | <11 (<15.7%) *^b^* |
| Surgery |  |  |
| No | 120 (71.4%) | 55 (78.6%) |
| Yes | 48 (28.6%) | 15 (21.4%) |
| Marital Status |  |  |
| Single/widowed | 60 (35.7%) | 14 (20.0%) |
| Married/domestic partner | 108 (64.3%) | 56 (80.0%) |
| Low-Income Subsidy Enrollment |  |  |
| No | 149 (88.7%) | >59 (>84.3%) *^b^* |
| At Least 1 Month | 19 (11.3%) | <11 (<15.7%) *^b^* |
| County-level Median Household Income ($) |  |  |
| <55,000 | 44 (26.2%) | 17 (24.3%) *^b^* |
| 55,000 – 64,999 | 19 (11.3%) |  |
| 65,000 – 74,999 | 39 (23.2%) | 19 (27.1%) |
| >75,000 | 66 (39.3%) | 34 (48.6%) |
| Geographic Region |  |  |
| Midwest | 15 (8.9%) | <11 (<15.7%) *^b^* |
| Northeast | 31 (18.5%) | >11 (>15.7%) *^b^* |
| South | 38 (22.6%) | 11 (15.7%) |
| West | 84 (50.0%) | 37 (52.9%) |

*^a^* PD-1 inhibitor includes nivolumab or pembrolizumab.

*^b^* Per SEER-Medicare confidentiality policies, cell sizes <11 have been masked.

Note: race and ethnicity are not included in this table due to the majority being NH White.

**Supplemental Table 4**. Number and location of Joinpoints in the final JPSurv models and average absolute change in 1-year and 5-year cause-specific survival with 95% confidence interval (CI).

| **Age** | **Stage** | **Number of Joinpoints** | **No. Alive at Start** | **Start Year** | **End Year** | **5-year Average Absolute Change (%)** | **5-year 95% CI** | **5-year Significance** | **1-year Average Absolute Change (%)** | **1-year 95% CI** | **1-year Significance** |
| --- | --- | --- | --- | --- | --- | --- | --- | --- | --- | --- | --- |
| ≥66 years | 3 | 1 | 5,630 | 2004 | 2013 | 0.8 | 0.10-1.50 | Increasing | 0.27 | 0.04-0.50 | Increasing |
|  |  |  |  | 2013 | 2019 | **3.72** | **2.81-4.62** | Increasing | **1.02** | **0.74-1.30** | Increasing |
| ≥66 years | 4 | 1 | 3,999 | 2004 | 2010 | 0.01 | -0.79-0.82 | n.s. | 0.02 | -1.00-1.03 | n.s. |
|  |  |  |  | 2010 | 2019 | **1.67** | **1.14-2.21** | Increasing | **1.78** | **1.19-2.38** | Increasing |
| <66 years | 3 | 1 | 11,056 | 2004 | 2011 | 0.16 | -0.37-0.69 | n.s. | 0.03 | -0.07-0.13 | n.s. |
|  |  |  |  | 2011 | 2019 | **2.03** | **1.62-2.44** | Increasing | **0.36** | **0.28-0.45** | Increasing |
| <66 years | 4 | 1 | 5,092 | 2004 | 2010 | 0.76 | 0.04-1.47 | Increasing | 0.87 | 0.05-1.68 | Increasing |
|  |  |  |  | 2010 | 2019 | **2.16** | **1.67-2.65** | Increasing | **1.99** | **1.51-2.47** | Increasing |

n.s. = not significant.
